# Editorial: Current concepts and recent advances on pediatric appendicitis

**DOI:** 10.3389/fped.2022.1111122

**Published:** 2023-01-04

**Authors:** Janne S. Suominen, Ramon R. Gorter, Jan F. Svensson

**Affiliations:** ^1^ Department of Pediatric Surgery, Helsinki University Hospital, University of Helsinki, Helsinki, Finland; ^2^ Department of Pediatric Surgery, Emma Children’s Hospital, Amsterdam University Medical Centers, University of Amsterdam, Amsterdam, Netherlands; ^3^ Department of Pediatric Surgery, Karolinska University Hospital, Karolinska Institutet, Stockholm, Sweden

**Keywords:** appendicitis, appendicitis - diagnosis, periappendiceal abscess, simple appendicitis, complicated appendicitis

**Editorial on the Research Topic** Current concepts and recent advances on pediatric appendicitis

Acute appendicitis is one of the most common pediatric surgical emergencies with a lifetime risk of approximately 8%, peaking between 10 and 15 years of age. Although it is a common illness, the exact pathophysiology of acute appendicitis remains somewhat unclear. Historically, acute appendicitis was considered to be an irreversible progressive disease starting with mild inflammation, but it will progress to necrosis, perforation and peritonitis over time. Early intervention (removal of the appendix) was therefore warranted. Recently, we rather endorse the idea that two different forms of acute appendicitis exist: uncomplicated (or simple) and complicated (or complex) appendicitis. Each form has its own clinical presentation, epidemiology, most likely pathophysiology, and also maybe treatment strategy. As both diagnostic work-up and treatment of acute appendicitis can be challenging, particularly in the pediatric population, further research on this topic is essential to improve the clinical care of these patients.

This special issue of *Frontiers in Pediatrics*: “Current concepts and recent advances on pediatric appendicitis” consists of four articles focusing on diagnostics, treatment, and pathophysiology of acute appendicitis. These articles have been selected as all of them contribute to the ongoing improvement of both clinical care as well as research in pediatric patients with appendicitis.

**Silva et al*.*** investigated the agreement between histopathological and intraoperative classifications and evaluated the predictive potential of these classifications for the outcome. The agreement was considered moderate and intraoperative classification was able to predict the postoperative clinical outcomes.

Over the past few years several biomarkers obtained from preoperative serum samples have been proposed as predictors of clinical outcomes. **Gudjonsdottir et al*.*** evaluated differences in serum concentrations of IgE and Th2-associated interleukins (IL) in children with uncomplicated and complicated appendicitis. High levels of IL-13 were found to be associated with an increased risk of complicated appendicitis.

Abscess formation after appendectomy is a rather typical postoperative complication. **van Amstel et al*.*** performed a systematic review on this topic and went through their own clinical data. Non-invasive treatment of medium sized abscesses seemed to be safe and effective. They proposed a new standardized treatment protocol for post-appendectomy abscesses in pediatric patients with aim to reduce the heterogeneity in clinical care for these patients. In general, such treatment protocols are useful to provide guidance to surgeons that encounter this “feared” complication. Even in case surgeons have less experience in treating this complication in this vulnerable patient group, tools from this treatment protocol could help surgeons to choose the optimal treatment strategy. Such treatment protocols can be set up for other complications or specific elements for the diagnostic workup or treatment of appendicitis as well. However, evaluation of such new treatment protocols after their implementation in daily practice is essential. Not only in the departments they were derived from, but more importantly in other departments to evaluate their external validity and generalizability.

**Reismann** summarized in his review the current understanding on epidemiology of acute appendicitis under Covid-19 pandemic.

These articles can be the base for further research and the optimal treatment of acute uncomplicated (simple) appendicitis in the pediatric population is under vigorous investigation. Hopefully in the next couple of years, the results of the ongoing randomized controlled trials on this topic in the pediatric population will be published. Essential for the success of non-operative treatment strategy is the accurate selection of children with uncomplicated appendicitis. To achieve accurate selection, the exact pathogenesis between uncomplicated and complicated appendicitis needs to be elucidated, and there is still work for better diagnostics as well. Understanding why one child develops an uncomplicated appendicitis while the other develops a complicated one will help us in identifying the most specific biomarkers that can be used in the near future for accurate selection. Integration of novel techniques (proteomics, metabolomics, and artificial intelligence) may even accelerate it.

We hope readers enjoy themselves with this special issue on pediatric appendicitis.

